# Tested and reported executive problems in children and youth epilepsy

**DOI:** 10.1002/brb3.971

**Published:** 2018-04-16

**Authors:** Erik Hessen, Kristin Å. Alfstad, Halvor Torgersen, Morten I. Lossius

**Affiliations:** ^1^ Division of Clinical Neuroscience National Centre for Epilepsy Oslo University Hospital Oslo Norway; ^2^ Department of Neurology Akershus University Hospital Lørenskog Norway; ^3^ Department of Psychology University of Oslo Oslo Norway; ^4^ University of Oslo Oslo Norway

**Keywords:** BRIEF, children, epilepsy, executive function, youth

## Abstract

**Objectives:**

Executive problems in children and youth with epilepsy influence their ability to handle important aspects of daily life activities. The present study sought to explore factors associated with executive problems for patients with epilepsy in this age group.

**Methods:**

The cohort consisted of 97 consecutive patients at the National Centre for Epilepsy in Norway, aged 10–19 years, with focal or genetic generalized epilepsy. All underwent tests of executive functions (D‐KEFS), the Behavior Rating Inventory for Executive Function (BRIEF), and screening for psychiatric symptoms, using the Strengths and Difficulties Questionnaire (SDQ).

**Results:**

Parent‐reported cognitive executive dysfunction (BRIEF, Metacognitive Index) was the strongest independent predictor for tested executive dysfunction and vice versa. Furthermore, male gender correlated strongest with parent‐reported behavioral regulation problems (BRIEF, Behavioral Regulation Index) along with borderline/pathological score on the SDQ and parent‐reported cognitive executive dysfunction.

**Conclusions:**

A strong association between parent‐reported cognitive executive dysfunction and tested executive dysfunction was found. Male gender correlated strongest with parent‐reported behavioral regulation problems. The latter was probably related to a higher frequency of symptoms associated with psychopathology among the boys than the girls. The frequency of executive deficits according to the different modes of measurement varied from 16% to 43%, suggesting that they capture different aspects of behavior under the executive umbrella.

## 1 INTRODUCTION

Children with epilepsy (CWE) have an increased prevalence of cognitive and executive problems (Berg et al., 2008; Fastenau et al., 2009; Hoie, Mykletun, Waaler, Skeidsvoll, & Sommerfelt, 2006; Hoie et al., 2005; Rantanen, Nieminen, & Eriksson, 2010). One population‐based study with emphasis on executive functions (EF) found that the patient group performed poorer on seven out of eight EF measures compared with the healthy comparison group (Hoie et al., 2006), and that executive problems contributed to school difficulties beyond intellectual dysfunction. EF includes behaviors necessary for successful coping with everyday challenges, such as initiation, planning, organization, purposive actions, self‐monitoring, and self‐regulation (Lezak, Howieson, Bigler, & Tranel, 2012). Thus, identification of factors associated with executive dysfunction in CWE is of importance for development of preventive measures and interventions to improve social and academic function.

Studies often define EF based on neuropsychological tests measuring aspects of these functions. Poor scores on EF tests have been associated with epilepsy‐related variables that also have shown association with other cognitive deficits: intellectual dysfunction (Hoie et al., 2005, 2006), psychiatric diagnosis (Alfstad et al., 2016), early seizure debut (Alfstad et al., 2016), integrity of corpus callosum and frontostriatal connections (Hermann, Hansen, Seidenberg, Magnotta, & O'Leary, 2003; Riley, Moore, Cramer, & Lin, 2011), different epilepsy syndromes (Bell, Lin, Seidenberg, & Hermann, 2011; Caplan et al., 2008; Kavros et al., 2008; Meeren, Pijn, EL Van, Coenen, & Lopes da Silva, 2002; Pascalicchio et al., 2007), high seizure frequency (Hoie et al., 2006) and antiepileptic drugs (Aldenkamp et al., 1993; Hessen, Lossius, Reinvang, & Gjerstad, 2006, 2007a, 2007b).

As the concept of EF largely pertains to everyday functioning (Lezak et al., 2012), one might question whether neuropsychological test performance validly captures this kind of function. The Behavior Rating Inventory of Executive Function (BRIEF) (Gioia, Isquith, Guy, & Kenworthy, 2000), a standardized parent‐rated measure assessing eight domains of EF, represents an effort to assess both behavioral and cognitive aspects of EF in everyday life. The BRIEF has been used to assess EF in several pediatric samples, including traumatic brain injury (Mangeot, Armstrong, Colvin, Yeates, & Taylor, 2002; Vriezen & Pigott, 2002), attention‐deficit–hyperactivity disorder (ADHD) (Jarratt, Riccio, & Siekierski, 2005), hydrocephalus (Mahone, Zabel, Levey, Verda, & Kinsman, 2002), and autism (Gilotty, Kenworthy, Sirian, Black, & Wagner, 2002; Gioia, Isquith, Kenworthy, & Barton, 2002). In pediatric epilepsy populations, poor scores on the BRIEF have shown association with everyday executive dysfunction (Campiglia et al., 2014) and poor health‐related quality of life (Sherman, Slick, & Eyrl, 2006). Slick, Lautzenhiser, Sherman, and Eyrl (2006) found that a substantial proportion of children with intractable epilepsy display significant EF deficits measured by BRIEF and called for research into the relationship of BRIEF scores to other measures of EF in children with epilepsy to further clarify its clinical utility. Only a few studies have adressed the relationship between tested and reported information about EF in children with epilepsy.

Parrish et al. (2007) looked at the BRIEF, completed by parents, and test results from aspects of the Delis‐Kaplan Executive Function System (D‐KEFS) and found in newly diagnosed children with epilepsy characterized by good seizure control that the two modes of measurement were significantly correlated on Metacognition Index, but not on the Behavioral Regulation Index. In a study by MacAllister et al. (2012), similar correlation between tested and reported EF deficits was not found when EF was tested with the Tower of London test (Anderson, Anderson, & Lajoie, 1996). They also found that the Tower of London performance but not ratings on the BRIEF could be predicted by epilepsy‐related variables, and discussed possible differences in the validity of these two modes of measuring EF.

Inconsistent findings between tested and reported executive deficits in children with epilepsy call for further investigation. Thus, the present study sought to analyze epilepsy‐related correlates to tested as well as reported behavioral and cognitive executive problems in children and youth referred to a tertiary epilepsy center. Based on referenced findings, we expected to find (Hoie et al., 2005) that tested executive problems would be associated with early seizure debut and reported everyday cognitive executive problems (Hoie et al., 2006), that behavioral aspects of reported everyday executive problems would be best correlated to psychiatric symptoms, and (Berg et al., 2008) that reported everyday cognitive executive problems both would be correlated to tested executive problems and to psychiatric symptoms.

## 2 METHODS

### 2.1 Patient inclusion and clinical data

**Figure 1:**
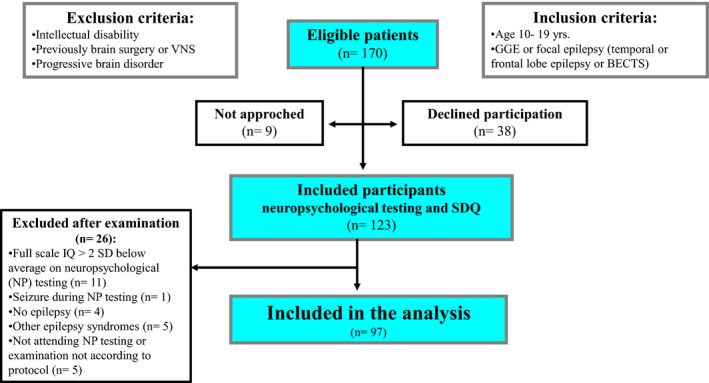
Flow chart, inclusion and exclusion criteria

Patients between 10 years and 19 years hospitalized at The National Centre for Epilepsy, the only tertiary epilepsy center in Norway, were included consecutively from January 2012 to June 2014 (Figure 1). Informed written consent was obtained from parents or participants of legal age. The study was approved by the Regional Ethics Committee (2011/1636/REK).

A detailed description of the inclusion and exclusion criteria in this study has been described elsewhere (Alfstad et al., 2016). All the patients underwent a 24‐hr EEG registration that was interpreted by a neurologist experienced in neurophysiology. The patients were assessed with MRI except those with benign childhood epilepsy with centrotemporal spikes (BECTS) or genetic generalized epilepsies (GGE). Two experienced neurologists classified the cases independently according to the International League Against Epilepsy (ILAE) classification of seizures and epilepsies (Berg et al., 2010). For non‐consensus or difficult cases, an experienced child neurologist, blinded to prior evaluations, reviewed the cases until consensus was reached.

The focal epilepsies were classified as temporal lobe (TLE), frontal lobe (FLE), BECTS, or focal epilepsy from anterior brain regions, but difficult to subclassify (focal epilepsy nos). The genetic generalized epilepsies (GGE) included were generalized epilepsy with febrile seizures plus (GEFS+), childhood absence epilepsy (CAE), juvenile absence epilepsy (JAE), juvenile myoclonic epilepsy (JME), generalized tonic clonic seizures (GTC) only, and GGE not otherwise specified (nos). Patients with Full Scale IQ below 70 and focal epilepsies from the posterior regions (parietal or occipital) were excluded.

### 2.2 Behavioral evaluation

The Strengths and Difficulties Questionnaire (SDQ) (Goodman, 2001) is a 25‐item brief behavioral screening questionnaire that was completed independently both as self‐report by the patients and by the parents. The questionnaire includes five subscales, covering emotional, conduct, hyperactivity, and peer relationships, as well as prosocial behavior. The items are rated “not true” (0), “somewhat true” (1), or certainly true” (2), resulting in a total difficulties score from 0 to 40. According to available norms (Goodman, 2001), the total scores were classified as normal 0–15, borderline 16–19, or abnormal >19 in the self‐report and correspondingly in the parent report 0–13, 14–16 and >16. Other research groups have previously used this methodological approach to classify scores on the SDQ (Alfstad et al., 2016; Heiervang et al., 2007).

The Behavior Rating Inventory of Executive Function (BRIEF) (Gioia et al., 2000), a standardized 86‐item parent‐rated measure, was employed to assess both behavioral and cognitive aspects of EF in everyday life. The subscales of inhibit, shift, and emotional control make up the summary Behavioral Regulation Index (BRI), while the Metacognition Index (MCI) is comprised of initiate, working memory, plan/organize, organizations, and monitor subscales. Scores are standardized based on a normal distribution with a mean of 50 and *SD* of 10. We examined scores on both the BRI and MCI and according to the manual (Gioia et al., 2000) *T*‐scores ≥ 65 were considered abnormal.

### 2.3 Neuropsychological assessment

General cognitive ability was measured by the Wechsler Abbreviated Scale of Intelligence (WASI) (Wechsler, 1999). Aspects of EFs were assessed by subtests from D‐KEFS (Delis, Kaplan, & Kramer, 2001): divided attention (Number‐Letter Switching), word fluency (Letter Fluency), and selective attention/response inhibition (Color Word Interference Test). These subtests are commonly used in the clinical and research literature of EF, and the D‐KEFS technical manual specifies that these tests can be used as stand‐alone measures of EF or part of a larger battery (Delis et al., 2001). All the raw scores were converted to *T*‐scores. A composite Executive Score was computed based on average *T*‐scores for three executive subtests. For the cutoff between normal and abnormal neuropsychological function, we used 1.5 Standard Deviations (*SD*) below normative mean (IQ ≤ 78 (WASI) and *T*‐score ≤ 35 (D‐KEFS)). We have previously employed the same composite score with the same cutoff criterion in a study of associations between executive dysfunction and psychiatric diagnosis in children with epilepsy (Alfstad et al., 2016).

### 2.4 Statistical analysis

First, descriptive statistics of the demographic, clinical, behavioral, and cognitive characteristics of the patient population was computed. Group comparisons were tested using Pearson's Chi‐square for categorical variables and independent sample *t* tests for continuous variables. Odds ratios for occurrence of tested and reported executive problems were estimated using logistic regression analysis. Univariate analyses were first performed for dichotomized variables reported in Tables 2, 3, 4, and significant factors were included in the multivariate analysis. Results of multivariate analysis are presented with odds ratios (OR) with 95% confidence intervals (CI) and *p* values. All tests were two‐sided and performed at a 5% significance level. The statistical Package for Social Sciences (spss version 24) was used.

## 3 RESULTS

Demographical, epilepsy‐related, and behavioral background characteristics are provided in Table 1. Table 2 shows that patients scoring below and above cutoff on the Executive Score are similar on all the demographical and epilepsy‐related variables and significantly different on all the cognitive and behavioral measures with poorest results for the group with Executive Score below cutoff. Table 3 shows similar results for all the cognitive and behavioral measures for the groups obtaining abnormally high score versus normal score on the Metacognitive Index. Additionally, it was found that patients with abnormally high score on the Metacognitive Index had significantly earlier seizure debut (7.0 vs. 8.7 years, *p* = .03) than those with scores in the normal range. In Table 4, comparison between patients with abnormally high versus normal score on the Behavioral Regulation Index shows that boys with epilepsy are significantly more likely to have behavioral regulation problems than girls. Other significant risk factors for obtaining high score on this index were early seizure debut, younger age at examination, high score both on SDQ, and Metacognitive Index as well as poor Executive Score. Of note, executive deficits according to the three different modes of measurement was found in 16% of the group when measured with neuropsychological tests, 43% of the group when measured by parent report of everyday cognitive executive problems in daily life, and 28% of the group when measured by parent report of everyday behavioral regulation difficulties. Difficulties according to the latter measurement method were primarily evident in boys, 51% in boys versus 8% in girls.

**Table 1:** Background characteristics of participants (*n* = 97)

Variables	Frequency *n* (%)
Gender male/female	44 (45.0)/53 (55.0)
Age epilepsy debut, mean (range, *SD*)	8.0 (<1–16 years, 4.0)
Age at examination, mean (range, *SD*)	14.0 (10–19 years, 2.4)
Epilepsy duration, mean (range, *SD*)	6.0 (1–16 years, 3.9)
Epilepsy syndrome	
TLE	14 (14.4)
FLE	20 (20.6)
JME	4 (4.1)
BECTS	12 (12.4)
Focal epilepsy	1 (1.0)
Absence epilepsy	18 (18.6)
Other GGE	28 (28.9)
Etiology	
Structural pathology	17 (17.5)
Genetic	46 (47.4)
Unknown	34 (35.1)
Seizure frequency	
No seizures last 6 months	35 (36.1)
No GTCs last 6 months	54 (55.7)
AED	
2 or 3 AED	40 (41.2)
1 AED	51 (52.6)
No AED	6 (6.2)
EEG	
No epileptiform activity	28 (28.9)
Cognitive and behavioral variables	
Full‐scale IQ, mean (range, *SD*)	93.7 (70–125, 12.1)
Executive *T*‐score, mean (range, *SD*)	43.8 (24–65, 8.5)
SDQ raw‐score parent report, mean (range, *SD*)	13.8 (0–29, 7.5)
BRIEF Behavioral Regulation Index (BRI) *T*‐score, mean (range, *SD*)	58.4 (37–95, 13.9)
BRIEF Metacognitive index (MI) *T*‐score, mean (range, *SD*)	61.6 (36–85, 12.8)

AED, antiepileptic drug; BECTS, benign childhood epilepsy with centrotemporal spikes; FLE, frontal lobe epilepsy; GGE, genetic generalized epilepsy; GTC only, generalized tonic clonic seizures only; JME, juvenile myoclonic epilepsy; nos, not otherwise specified; TLE, temporal lobe epilepsy.

**Table 2:** Low Executive Score (*T*‐score ≤ 35) versus normal Executive Score (*T*‐score > 35) (chi‐square and independent *t* test, *n* = 96)

Variables	Low Executive Score, *n* = 15 (16%) *n* (%)/mean (*SD*)	Normal Executive Score, *n* = 81 (84%) *n* (%)/mean (*SD*)	*p* Value
Boys	8 (53.3)	35 (43.2)	ns
Girls	7 (46.7)	46 (56.8)	ns
Age at epilepsy debut	8.3 (4.6)	8.0 (3.8)	ns
Age at examination	14.5 (2.5)	14.0 (2.4)	ns
Epilepsy duration	6.1 (4.2)	6.0 (3.9)	ns
Focal epilepsy	6 (40.0)	41 (50.6)	ns
GGE	9 (60.0)	40 (49.4)	ns
No GTCs last 6 months	7 (46.7)	44 (54.3)	ns
MRI structural lesion	2 (13.3)	20 (24.7)	ns
≥ 2AED (polytherapy)	9 (60.0)	31 (38.3)	ns
EEG no epileptiform activity	8 (53.3)	23 (28.4)	ns
Full‐scale IQ	86.3 (8.1)	95.1 (12.3)	.002
SDQ score: parent report, raw score	18.4 (6.0)	12.7 (7.4)	.008
Behavioral Regulation Index, *T*‐score	66.7 (13.8)	56.4 (12.9)	.006
Metacognitive Index, *T*‐score	72.0 (8.4)	59.4 (12.5)	.001

AED, antiepileptic drug; GGE, genetic generalized epilepsy.

**Table 3:** High Score on the Metacognitive Index (MI) (*T*‐score ≥ 65) versus normal Score on the MI (*T*‐score < 65) (chi‐square and independent *t* test, *n* = 93)

Variables	High Score on MI, *n* = 40 (43%) *n* (%)/mean (*SD*)	Normal Score on MI, *n* = 53 (57%) *n* (%)/mean (*SD*)	*p* Value
Boys	21 (52.5)	21 (39.6)	ns
Girls	19 (47.5)	32 (60.4)	ns
Age at epilepsy debut	7.0 (4.0)	8.7 (3.7)	.03
Age at examination	13.6 (2.2)	14.1 (2.4)	ns
Epilepsy duration	6.6 (4.3)	5.5 (3.3)	ns
Focal epilepsy	17 (42.5)	28 (52.8)	ns
GGE	23 (57.5)	25 (47.2)	ns
No GTCs last 6 months	19 (47.5)	31 (58.5)	ns
MRI structural lesion	8 **(**20.0)	13 (24.5)	ns
≥ 2AED (polytherapy)	15 (37.5)	22 (41.5)	ns
EEG no epileptiform activity	9 (22.5)	17 (32.1)	ns
Full‐scale IQ	90.0 (10.9)	96.7 (12.4)	.008
SDQ score: parent report, raw score	18.9 (6.6)	9.8 (5.5)	.001
Behavioral Regulation Index, *T*‐score	67.6 (13.5)	51.4 (9.7)	.001
Executive Score, *T*‐score	39.8 (8.6)	46.5 (7.5)	.001

AED, antiepileptic drug; GGE, genetic generalized epilepsy.

**Table 4:** High Score on the Behavioral Regulation index (BRI) (*T*‐score ≥ 65) versus normal Score on the BRI (*T*‐score < 65) (chi‐square and independent *t* test, *n* = 94)

Variables	High Score on BRI, *n* = 26 (28%) *n* (%)/mean (*SD*)	Normal Score on BRI, *n* = 68 (72%) *n* (%)/mean (*SD*)	*p* Value
Boys	22 (84.6)	21 (30.9)	.001
Girls	4 (15.4)	47 (69.1)	.001
Age at epilepsy debut	6.1 (3.6)	8.6 (3.9)	.008
Age at examination	12.9 (2.0)	14.3 (2.4)	.007
Epilepsy duration	6.7 (4.2)	5.8 (3.8)	ns
Focal epilepsy	13 (50.0)	32 (47.1)	ns
GGE	13 (50.0)	36 (52.9)	ns
No GTCs last 6 months	14 (53.8)	36 (52.9)	ns
MRI structural lesion	6 (23.1)	15 (22.1)	ns
≥ 2AED (polytherapy)	9 (34.6)	29 (42.6)	ns
EEG no epileptiform activity	5 (19.2)	21 (30.9)	ns
Full‐scale IQ	91.1 (10.9)	94.6 (12.4)	ns
SDQ score: parent report, raw score	22.8 (5.1)	10.3 (4.8)	.001
Metacognitive Index, *T*‐score	73.4 (8.9)	57.1 (11.1)	.001
Executive Score, *T*‐score	39.6 (9.2)	45.1 (7.8)	.005

AED, antiepileptic drug; GGE, genetic generalized epilepsy.

### 3.1 Associations between tested and reported cognitive and behavioral executive problems

#### 3.1.1 Executive Score

Univariate logistic regression analysis showed that high score on the Metacognitive Index (OR = 12.8, CI 2.7–60.8, *p* = .001), borderline/pathological score on SDQ (OR = 8.1, CI 1.7–38.6, *p* = .009), and high score on the Behavioral Regulation Index (OR = 4.1 CI 1.3–12.9, *p* = .016) were significantly associated with poor Executive Score (Table 5). Only high score on the Metacognitive Index factors remained significant in the multivariate analysis (OR 14.2, CI 1.5–128.4, *p* = .01).

**Table 5:** Univariate and multivariate regression analyses (dependent variable: Low Executive Score (*T*‐score ≤ 35))

Variable	Univariate analysis		Multivariate analysis	
	OR (95% CI)	*p* Value	OR (95% CI)	*p* Value
Metacognitive Index ≥ *T*‐score 65	12.8 (2.7–60.8)	.001	14.2 (1.5–128.4)	.01
Borderline/pathological score on SDQ	8.1 (1.7–38.6)	.009	2.8 (0.4–19.2)	ns
Behavioral Regulation Index ≥ *T*‐score 65	4.1 (1.3–12.9)	.016	1.1 (0.3–5.0)	ns

#### 3.1.2 Behavioral Regulation Index

Univariate logistic regression analysis showed that Male gender (OR = 12.3, CI 3.8–40.2, *p* = .001), borderline/pathological score on SDQ (OR = 23.4, CI 5.0–108.8, *p* = .001), high score on the Metacognitive Index (OR = 10.6 CI 3.5–32.2, *p* = .001), epilepsy debut before 8 years (OR=2.7 CI 1.1–9.9, *p* = .039), and poor Executive Score (OR = 4.1 CI 1.3–12.9, *p* = .016) were significantly associated with high score on the Behavioral Regulation Index (Table 6). Only three of these variables remained significant in the multivariate analysis; Male gender (OR 14.8, CI 3.2–69.3, *p* = .001), borderline/pathological score on the SDQ (OR 7.7, CI 1.3–46.4, *p* = .026), and high score on the Metacognitive Index (OR = 5.6 CI 1.1–28.4, *p* = .036).

**Table 6:** Univariate and multivariate regression analyses (dependent variable: Behavioral Regulation Index ≥ *T*‐score 65)

Variable	Univariate analysis		Multivariate analysis	
	OR (95% CI)	*p* Value	OR (95% CI)	*p* Value
Male gender	12.3 (3.8–40.2)	.001	14.8 (3.2–68.3)	.001
Borderline/pathological score on SDQ	23.4 (5.0–108.8)	.001	7.7 (1.3–46.4)	.026
Metacognitive Index ≥ *T*‐score 65	10.6 (3.5–32.2)	.001	5.6 (1.1–28.4)	.036
Epilepsy debut before 8 years	2.7 (1.1–9.9)	.039	1.9 (0.5–7.8)	ns
Low Executive Score (*T*‐score ≤ 35)	4.1 (1.3–12.9)	.016	1.5 (0.3–8.4)	ns

#### 3.1.3 Metacognitive Index

Univariate logistic regression analysis showed that poor Executive Score (OR = 12.8, CI 2.7–60.1, *p* = .001), borderline/pathological score on SDQ (OR = 10.6, CI 3.5–32.2, *p* = .001), high score on the Behavioral Regulation Index (OR = 10.6 CI 3.5–32.2, *p* = .001), and epilepsy debut before 8 years (OR=2.5 CI 1.1–5.7, *p* = .035) were significantly associated with high score on the Metacognitive Index (Table 7). Only two of these factors remained significant in the multivariate analysis; poor Executive Score (OR 18.5, CI 1.9–180.0, *p* = .01) and borderline/pathological score on the SDQ (OR 3.7, CI 1.1–11.9, *p* = .03).

**Table 7:** Univariate and multivariate regression analyses (dependent variable: Metacognitive Index ≥ *T*‐score 65)

Variable	Univariate analysis		Multivariate analysis	
	OR (95% CI)	*p* value	OR (95% CI)	*p* value
Low Executive Score (*T*‐score ≤ 35)	12.8 (2.7–60.1)	.001	18.5 (1.9–180.0)	.01
Borderline/pathological score on SDQ	10.6 (3.5–32.2)	.001	3.7 (1.1–11.9)	.03
Behavioral Regulation Index ≥ *T*‐score 65	10.6 (3.5–32.2)	.001	3.6 (0.9–28.4)	ns
Epilepsy debut before 8 years	2.5 (1.1–5.7)	.035	2.0 (0.7–6.1)	ns

## 4 DISCUSSION

The main findings of this study are that parent‐reported everyday cognitive executive dysfunction best correlated with tested executive dysfunction and vice versa, that tested executive dysfunction was best correlated with parent‐reported everyday cognitive executive dysfunction. Furthermore, male gender showed the strongest association with parent‐reported everyday behavioral regulation problems along with borderline/pathological score on the SDQ and parent‐reported everyday cognitive executive dysfunction. Borderline/pathological score on the SDQ was also significantly associated with parent‐reported everyday cognitive executive dysfunction. Executive deficits according to the three different modes of measurement was found in 16% based on tested executive dysfunction, 43% based on parent report of everyday cognitive executive problems, and 28% based on parent report of everyday behavioral regulation difficulties.

The strong association between parent‐reported everyday cognitive executive and tested executive dysfunction in this group of children with complex and chronic epilepsy is similar to what Parrish et al. (2007) found in children with newly diagnosed epilepsy and with good seizure control. This suggests a generalizability of findings for these specific measurement methods across epilepsy characteristics. However, the findings do not match with a recent study by MacAllister et al. (2012) who in children with epilepsy found no correlation on parent report of everyday executive dysfunction, employing the BRIEF, and the Tower of London test (Anderson et al., 1996), which is a measure of EF. This finding fits well with studies that evaluate the association between reported and tested EF in children with other neurologic conditions. For instance, Anderson, Anderson, Northam, Jacobs, and Mikiewicz (2002) found hardly any correlation between parent‐reported everyday executive problems measured with BRIEF in a group of children with diverse neurologic disorders (phenylketonuria, hydrocephalus, and frontal focal lesion) and tested executive dysfunction. Both MacAllister et al. (2012) and others indicate that reported everyday behavioral and tested measures appear to tap different constructs within the executive domains, suggesting that the BRIEF reflects more daily “real‐life” behavior, while performance on neuropsychological primarily predicts behavior in a controlled assessment setting.

Male gender showed by far the strongest association with high score on the Behavioral Regulation Index. Fifty‐one percent of the boys achieved scores in the abnormal range and only 8% of the girls, which means that the boys in this sample to a greater extent than the girls struggle with inhibition, mental flexibility, and emotional control. We have not found other studies in pediatric epilepsy populations with similar gender differences on the BRIEF Behavioral Regulation Index. In a previous study largely based on the same study population as the present paper Alfstad et al. (2016) found that significantly more boys than girls had a psychiatric diagnosis, primarily associated with ADHD and anxiety. The present study does not include formal psychiatric diagnosis, only a brief parent report of behavioral screening with the SDQ (Goodman, 2001) that has shown good ability to detect psychiatric problems (Goodman, Ford, Simmons, Gatward, & Meltzer, 2000), covering emotional aspects, conduct, hyperactivity and peer relationships, as well as prosocial behavior. In our sample, the BRIEF Behavioral Regulation Index and the SDQ score were highly correlated (*r* = .829, *p* = .001). The boys achieved a mean raw score on the SDQ of 17.0 and the girls a mean score of 11.0 (*p* = .001). Together this suggests that a high level of psychiatric problems in the boys group contributes to a high level of everyday behavioral executive dysfunction. Other studies have also suggested an association with behavioral/psychiatric problems and everyday executive problems in children with epilepsy (Baum et al., 2010; Ekinici, Titus, Rodopman, Berkem, & Trevathan, 2009). Employing the child behavior check‐list (CBCL) and neuropsychological testing, Baum et al. (2010) investigated a group of children with a first recognized seizure, and found a connection between executive problems and depressive and anxious mood symptoms (Baum et al., 2010).

Early seizure onset, defined as below mean for the group (below 8 years of age) was associated with higher and more pathological scores on both the Metacognitive Index and the Behavioral Regulation Index on the BRIEF, but not on the composite Executive Score. As mentioned above, the BRIEF correlates highly with SDQ, which is associated with psychiatric symptoms. Thus, this finding is probably in line with our previous finding of high association between early seizure debut and psychiatric comorbidity (Alfstad et al., 2016). Most previous studies have not found this association (Alfstad et al., 2011). The divergent findings may be related to a host of factors, including different study populations, different measurement methods and which cutoff for early versus late seizure debut that has been employed. The finding that tested EF was not associated with early seizure debut fits with findings, suggesting that early seizure debut is primarily associated with lower IQ in pediatric populations (Hoie et al., 2005). Other seizure variables, such as seizure frequency, showed no association with any of the three modes of measuring executive problems, employed in this study. AED monotherapy can have cognitive and executive side effects (Aldenkamp et al., 1993; Hessen et al., 2006, 2007a) and AED polytherapy may have an additional increased risk for cognitive side effects (Aldenkamp et al., 1993). However, in this study, we found no association between AEDs and EF, in particular AED polytherapy and executive deficits.

Executive function is an umbrella term comprising very different cognitive processes and behavioral competencies including initiation, planning, verbal reasoning, problem‐solving, the ability to sustain attention, resistance to interference, self‐monitoring, multitasking, cognitive flexibility, and the ability to deal with novelty (Lezak et al., 2012). To assess these functions, a wide range of tests, inventories, and tasks have been developed, under the same umbrella term, in countries with different cultures, developmental levels, and different languages, and also normed in unlike settings (Raymond, Chan, David Shum, Toulopoulou, & Chen, 2008). Based on this myriad of unlike measures and validation, it is not surprising that different studies often do not find similar associations between medical and psychological conditions and what is called EFs in a particular study. In this study, we employed three modes of measurement that despite significant associations obviously captures different function and behavior. This is evident when cutoff 1.5 *SD* from the normative mean characterizes 16% of the group based on neuropsychological tests, 43% of the group based on parent report of everyday cognitive executive problems in daily life, and 28% of the group based on parent report of everyday behavioral regulation difficulties in daily life. These very diverse findings based on measures that all are named as executive measures raises the question of whether the term EF may be too broad to be meaningful in many research and clinical settings.

Another limitation of the study is the recruitment bias. All patients were included consecutively while hospitalized at The National Centre for Epilepsy, that is, a tertiary epilepsy center. They may be representative for patients fulfilling our selection criteria, but not for children with epilepsy in a population‐based setting, as the majority of persons with epilepsy are well controlled, mostly seizure free (Kwan & Sander, 2004).

In conclusion, the main finding of this study was a strong association between parent‐reported everyday cognitive executive dysfunction and tested executive dysfunction. Furthermore, male gender showed the strongest association with parent‐reported everyday behavioral regulation problems, probably related to a higher frequency of symptoms associated with psychopathology among the boys than the girls. Despite strong associations between the modes of measurement, the frequency of executive deficits according to the three different modes varied much, from 16% to 43%, suggesting that they capture different aspects of behavior under the executive umbrella. These diverse findings based on measures that all are named as executive measures raise the question of whether the term EF may be too broad to be meaningful.

## ETHICAL APPROVAL

We confirm that we have read the Journal's position on issues involved in the ethical publication and affirm that this report is consistent with those guidelines.

## CONFLICT OF INTEREST

None of the authors has any conflict of interest related to this article to disclose.
